# Study on the Trend and Disease Burden of Injury Deaths in Chinese Population, 2004–2010

**DOI:** 10.1371/journal.pone.0085319

**Published:** 2014-01-17

**Authors:** Lijuan Zhang, Zhiqiang Li, Xucheng Li, Jie Zhang, Liang Zheng, Chenghua Jiang, Jue Li

**Affiliations:** Department of Prevention, Tongji University School of Medicine, Shanghai, China; Indian Institute of Toxicology Reserach, India

## Abstract

Injuries are a growing public health concern in China, accounting for more than 30% of all Person Years of Life Lost (PYLL) due to premature mortality. This study analyzes the trend and disease burden of injury deaths in Chinese population from 2004 to 2010, using data from the National Disease Surveillance Points (DSPs) system, as injury deaths are classified based on the International Classification of Disease-10^th^ Revision (ICD-10). We observed that injury death accounted for nearly 10% of all deaths in China throughout the period 2004–2010, and the injury mortality rates were higher in males than those in females, and higher in rural areas than in urban areas. Traffic crashes (33.79–38.47% of all injury deaths) and suicides (16.20–22.01%) were the two leading causes of injury deaths. Alarmingly, suicide surpassed traffic crashes as the leading cause of injury mortality in rural females, yet adults aged 65 and older suffered the greatest number of fatal falls (20,701 deaths, 2004–2010). The burden of injury among men (72.11%) was about three times more than that of women's (28.89%). This study provides indispensible evidence that China Authority needs to improve the surveillance and deterrence of three major types of injuries: Traffic-related injury deaths should be targeted for injury prevention activities in all population, people aged 65+ should be encouraged to take individual fall precautions, and prevention of suicidal behavior in rural females should be another key priority for the government of China.

## Introduction

Various types of injuries collectively are a growing public health issue which has become an important cause of death throughout the world. Injuries kill more than five million people worldwide in 2000, accounting for nearly one of every 10 deaths [1]. Around the world, about 16,000 people die every day as a result of an injury [2], [3]. Among the causes of injury are acts of violence, road traffic crashes, assault, drowning, falls and poisoning. The deaths caused by injury have a serious impact on the families and communities, where life is often irrevocably changed by these tragedies. According to the World Health Organization (WHO), over 90% of injury-related deaths occurred in low- and middle-income countries in 2004 [4].

China is a developing country with the largest population in the world. Meantime, China's rapid economic growth has been accompanied by substantial changes in modes of transport, lifestyle and so on, all of which cause many unexpected issues and problems. For instance, in the past two decades, the main transportation mode has changed from animal carts and bicycles to motor vehicles, it is estimated that about 55 thousand new motor vehicles are registered in China every day [5]. The population is ageing; as the proportion of the population aged 65 and above grew from 7.0% to 8.9% throughout the period of 2000–2010 [6]. Moreover, the gap between the rich and the poor is widening; by 2004, the gross domestic product per person in the richest province was 13 times greater than that in the poorest province [7]. All these contribute to the astonishing reality that injury has silently grown to be the fourth leading cause of death in China [8]. Injuries now are an additional public health threat in China, causing at least 800,000 deaths and 50 million non-fatal damages each year, of which 2.3 million cases lead to disability with varying degrees of severity [9], [10] and the hefty medical expenses that cost the taxpayers 65 billion RMB every year [11]. In addition, injury is also the leading cause of death in China from age 1–39 [12], causing annual loss of 12.6 million potentially productive years of life, a loss greater than for any disease group [13]. The estimated annual economic cost of injury is almost 4 times the total public health services budget in China. Considering the *status quo* of China's injury scenarios and its rapid societal change, the drive for injury prevention and safety promotion need to be further strengthened, and there is an urgent need for the development of a national injury prevention/safe community program.

This study was designed to look at the distribution and trend of injury deaths from 2004 to 2010 in China by criterions such as age, gender, urban/rural residence and regions. We further performed a detailed analysis of China-specific characteristics of the leading causes of injury-related mortality, providing some scientific basis for preventing injury and death incidences. The results will serve to national injury prevention strategies, and, hopefully, our conclusions will help push forward the movement of injury control in China.

## Results

### Basic Information of Injury Deaths in China, 2004–2010

In the DSPs system, the rates and proportions of death from injuries in China are categorized by gender, age and geographical region, respectively. In this investigation, among 73 million people investigated per year from 2004 to 2010, more than 400,000 people died of all causes every year except for the year 2006 (Table 1). The number of deaths caused by injuries accounted for about 10% of all deaths in China during the study period and the total number of injury deaths was 25,858–32,281 in rural areas and 8,645–11,815 in urban areas (2004–2010). The number of injury deaths among rural males was 18,029–22,195 persons/year, which was the highest among groups of different areas, followed by rural female and urban male groups. The number of injury deaths in urban female group was the lowest, 2,912–4,081 persons/year.

**Table 1:** Basic information of injury deaths in China, 2004–2010.

	Year						
	2004	2005	2006	2007	2008	2009	2010
Total people surveyed(*million)	71.17	71.49	73.78	71.48	75.14	75.67	75.67
Total number of death	430994	437490	347058	401008	424683	437550	453211
**Death caused by injury**	43979	43774	34503	39114	40581	40447	40321
**Urban**	11815	11493	8645	10459	10041	10597	10922
Male	7734	7573	5733	7069	6782	7181	7346
Female	4081	3920	2912	3390	3259	3416	3576
**Rural**	32164	32281	25858	28655	30540	29850	29399
Male	22195	22162	18029	20210	21290	21008	20862
Female	9969	10119	7829	8445	9250	8842	8537

### Demographic Trend of Injury Mortality Rates (/100,000/year) by Gender and Urban/Rural Residence in China, 2004–2010

**Figure 1:**
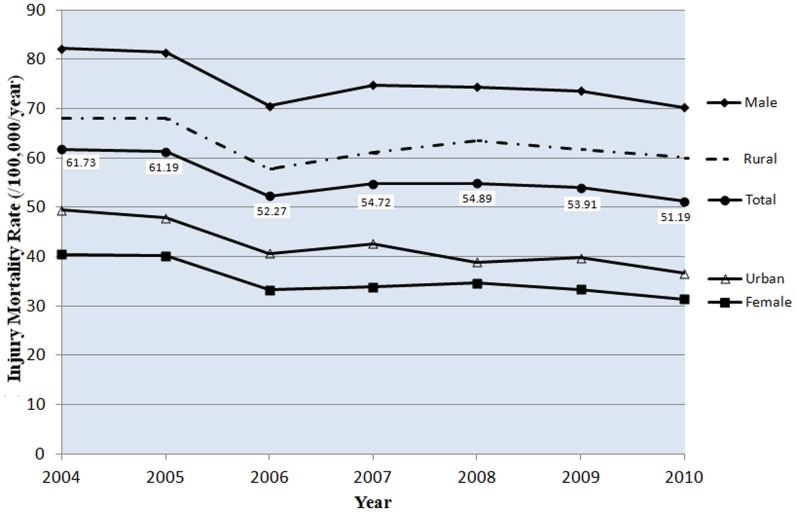
Demographic trend of injury mortality rates (/100,000/year) by gender and urban/rural residence in China, 2004–2010. The trends of injury mortality rates (/100,000/year) from 2004–2010 in males and females, urban and rural areas in China are showed.

The injury mortality rate in males was consistently high during the 7 years (Figure 1), in the range of 70.21–82.18 deaths per hundred thousand per year. This was much higher than the rate in females, more than twice as high (Table 2). The injury mortality rate in rural areas was also consistently high in the same time period (Figure 1), in the range of 60.06–68.03 deaths/100 000/year, about 1.37–1.64 times higher than the rate in urban areas (Table 2).

**Table 2:** Injury mortality rates (100, 000/year) by gender and urban/rural residence in China, 2004–2010.

		Year							Regression coefficient *B*	*t*	*p*-Value
		2004	2005	2006	2007	2008	2009	2010			
**Total**		61.79	61.23	52.27	54.72	54.89	53.91	51.19	−1.52	−3.04	0.029
**Gender**	Male	82.18	81.35	70.49	74.76	74.34	73.63	70.21	−1.70	−2.67	0.044
	Female	40.42	40.19	33.25	33.84	34.59	33.37	31.39	−1.41	−3.67	0.014
**Region**	**Urban**	49.44	47.88	40.60	42.61	38.83	39.75	36.63	−2.02	−5.10	0.004
	Male	63.77	62.21	53.23	56.83	51.91	53.28	48.74	−2.30	−4.52	0.006
	Female	34.68	33.14	27.67	28.00	25.46	25.91	24.26	−1.71	−0.93	0.002
	**Rural**	68.03	67.98	57.82	61.06	63.54	61.73	60.06	−1.10	−1.71	0.148
	Male	91.38	90.90	78.60	84.03	86.21	84.69	83.10	−1.06	−1.33	0.242
	Female	43.37	43.80	35.95	36.91	39.59	37.54	35.80	−1.13	−2.33	0.067

Linear regression analysis showed a gradual decline in both males and females during the study period, with the regression coefficient was −1.70 and −1.41, respectively (*p*<0.05). However, the differences were not statistically significant between the rates of rural male and female groups (*p*>0.05) (Table 2).

### The Proportion Dissection of Standardized Injury Mortality by Leading Causes in the Overall Injury Deaths in China, 2004–2010

The number one leading cause of death from injury was road traffic crashes, accounting for 33.79 to 38.47% of all injury deaths during the period 2004–2010 in China; the second was suicide, accounting for 16.20% to 22.01%; and the third was fall, accounting for 11.98% to 14.22%. These top three causes are jointly responsible for 61.97% to 74.82% of all injury deaths. Other leading causes, including drowning, poisoning and assault account about 17% of injury deaths. Proportions of all the leading causes in the gross injury deaths have not significantly changed throughout the study period (*p*>0.05) (Table 3).

**Table 3:** The proportion dissection of standardized injury mortality by leading causes in the overall injury deaths during 2004–2010 in China.

Cause	2004		2005		2006		2007		2008		2009		2010		*p*-value
	Rank	%	Rank	%	Rank	%	Rank	%	Rank	%	Rank	%	Rank	%	
Traffic	1	33.83	1	33.79	1	34.89	1	38.47	1	36.99	1	37.89	1	38.00	0.387
Suicide	2	22.01	2	20.39	2	19.34	2	18.40	2	17.28	2	17.09	2	16.20	0.205
Fall	3	11.98	3	12.86	3	14.34	3	13.57	3	13.49	3	13.95	3	14.22	0.698
Drowning	4	8.74	4	8.92	4	8.63	4	7.97	4	7.93	4	8.10	4	7.96	0.683
Poisoning	5	5.49	5	5.58	5	5.38	5	4.91	5	5.43	5	5.87	5	5.94	0.803
Assault	6	2.78	6	2.62	6	2.31	6	2.24	6	1.78	6	2.10	6	1.81	0.528
Others	-	15.17	-	15.84	-	15.21	-	14.44	-	17.10	-	15.00	-	15.87	0.875
Total	-	100.00	-	100.00	-	100.00	-	100.00	-	100.00	-	100.00	-	100.00	-

We further assessed the differences among different groups from respective urban/rural areas (Table 4). Overall, the top six leading causes were: traffic crashes, suicide, fall, drowning, poisoning and assault, both in urban and rural areas. Traffic crashes caused the most serious life loss with the highest standardized mortality rates, ranging 12.05–15.73 (deaths/100,000/year) in urban areas and 19.78–22.91 in rural areas. Surprisingly, there was a tendency of a decreasing demographic features from 2004 to 2010 for traffic, drowning, poisoning and assault deaths in urban areas, with the regression coefficients ranging from −5.60 to −3.08 (*p*<0.05). However, in rural areas, though a decreasing trend of suicide and assault was observed (*p*<0.05), flat trends of the standardized injury mortality rates generated by road traffic crashes, fall, drowning and poisoning only contributed a non-significant rate decrease throughout the period 2004–2010 (*p*>0.05).

**Table 4:** Standardized injury mortality rates (/100,000/year)^a^ by leading causes of death in China, 2004–2010.

Causes of death		Year							Regression coefficient *B*	*t*	*p*-Value
		2004	2005	2006	2007	2008	2009	2010			
**Urban**	Traffic	15.73	15.07	12.69	14.55	13.31	13.01	12.05	−0.52	−3.28	0.022
	Suicide	8.71	7.36	5.87	5.61	4.75	5.12	4.46	−0.66	−5.43	0.003
	Fall	6.17	6.17	5.61	5.33	5.02	5.46	5.52	−0.14	−2.31	0.069
	Drowning	3.45	3.34	2.73	2.81	2.43	2.57	2.30	−0.19	−5.60	0.003
	Poisoning	2.95	2.78	2.25	2.17	2.03	2.38	2.01	−0.14	−3.08	0.027
	Assault	1.93	1.61	1.30	1.18	0.85	0.06	0.73	−0.26	−4.50	0.006
**Rural**	Traffic	22.59	22.63	19.78	22.91	22.26	22.16	21.72	−0.04	−0.18	0.868
	Suicide	15.11	14.11	11.08	11.11	10.55	10.05	9.16	−0.95	−5.98	0.002
	Fall	6.92	7.46	7.02	7.00	6.95	7.60	7.33	0.05	0.98	0.370
	Drowning	6.42	6.49	5.35	5.12	5.30	5.55	5.25	−0.19	−2.42	0.060
	Poisoning	3.38	3.49	4.10	2.68	3.11	3.22	3.25	−0.07	−0.82	0.449
	Assault	1.56	1.55	1.11	1.19	0.98	1.21	1.05	−0.84	−2.78	0.039

^a^ Standardized to China standard population for the year 2000.

### Top Three Leading Causes of Average Injury Mortality by Urban/Rural Areas and Gender, China 2004–2010

The injury mortality rate was 62.89±3.90 in rural areas and 42.24±4.76/100,000/year in urban areas throughout the period 2004–2010. Injuries killed 85.55±4.48 rural residents per 100,000 people a year, approximately 1.5 times higher than the rate of urban residents. Traffic crashes was the number one leading cause of injury deaths in urban areas; followed by fall and suicide for urban males; for urban females, the suicide was the second leading cause of injury deaths, followed by falls. For rural males, traffic crashes killed 34.35±1.86 per 100,000 people a year, 1.5 times higher than the rate of urban males. Nevertheless, the rate of suicide surpassed the rate of road traffic crashes, claiming the number one leading cause of injury mortality in rural females (Table 5).

**Table 5:** Top three leading causes of average injury mortality rates (/100, 000/year) by urban/rural areas and gender in China, 2004–2010.

Rank	Urban		Rural	
	Male	Female	Male	Female
Total	42.24±4.76		62.89±3.90	
Gender	55.71±5.53	28.45±3.97	85.55±4.48	38.99±3.78
1	Traffic crashes	Traffic crashes	Traffic crashes	Suicide
	22.33±1.93	22.33±1.93	34.35±1.86	11.42±1.93
2	Falls	Suicide	Suicide	Traffic crashes
	8.06±0.16	6.48±1.68	13.08±2.07	10.43±0.46
3	Suicide	Falls	Falls	Falls
	7.59±1.40	5.84±0.52	9.54±0.27	5.87±0.34

### Demographic Trend of Injury Mortality Rate by Age in China, 2004–2010

**Figure 2:**
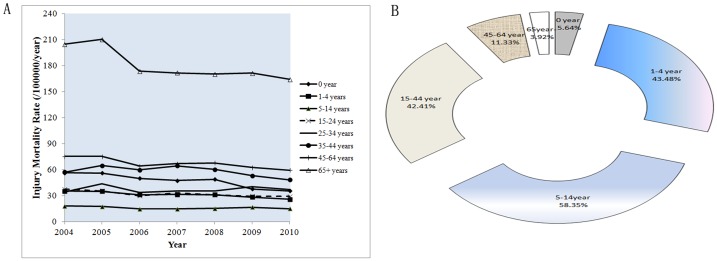
Demographic trends of the diverse injury mortality rates by the criterion of age. (A) The demographic trends of injury mortality by the criterion of age; (B) The proportional mortality rate caused by injury by the criterion of age.

We further assessed the demographic trends of the diverse injury mortality rates by the criterion of age (Figure 2A and B). The injury mortality rate was 14.68–18.05 deaths/100,000/year in 5–14 year-old age group, the lowest among all age groups. The rates were similar between 1–4 and 15–24 year age groups. During the study period, the injury mortality rate was 35.49–56.39 deaths/100,000/year in the zero-year-old age group and 33.79–44.00 deaths/100,000/year among 25–34 year-old adults. The rates then further increased to 48.33–64.64 and 59.47–75.38 deaths/100,000/year for 35–44 year-old and 45–64 year-old age groups, respectively. The injury mortality was 164.24–210.32 deaths/100,000/year in the 65+ year age group, the highest among all age groups (Figure 2A). Injury accounted for 58.35% of all deaths in the 5–14 year-old age group; meanwhile, injury deaths accounted for some 43.48% of all deaths among 1–4 year-old children and 42.41% among 15–44 year-old individuals, respectively. However, only about 3.92% of deaths were caused by injuries in the group of people aged 65 and beyond (Figure 2B).

### Top Six Causes of Death for Assorted Age Groups in China, 2004–2010

Drowning, road traffic crashes and falls represented the top three leading causes of injury deaths for children under one-year-old. For 1–4 year-olds, the three leading causes of injury deaths were traffic crashes, falls and drowning, while drowning surfaced as the leading cause of injury mortality for 5–14 year-old youths. For 15 to 64 year-old people, the number one leading cause of injury deaths was due to road traffic crashes. Starting at age 65, fall and suicide were the two most prominent causes of injury deaths (Table 6).

**Table 6:** Top six causes of death for assorted age groups in China, 2004–2010.

Rank	0 year	1–4 years	5–14 years	15–24 years	25–34 years	35–44 years	45–64 years	65+ years
**1**	Drowning	Traffic crashes	Drowning	Traffic crashes	Traffic crashes	Traffic crashes	Traffic crashes	Falls
	236	1484	5300	14949	17268	23388	30355	20701
**2**	Traffic crashes	Falls	Traffic crashes	Suicide	Suicide	Suicide	Suicide	Suicide
	193	497	3092	3612	5320	16627	15961	19857
**3**	Falls	Drowning	Falls	Drowning	Falls	Falls	Falls	Traffic crashes
	164	497	601	2897	2139	7742	8304	15275
**4**	Poisoning	Poisoning	Suicide	Falls	Poisoning	Drowning	Poisoning	Drowning
	101	272	440	1369	1866	6693	4957	4304
**5**	Assault	Assault	Poisoning	Poisoning	Drowning	Poisoning	Drowning	Poisoning
	73	12	402	1280	1650	6347	3486	3631
**6**	Suicide	Suicide	Assault	Assault	Assault	Assault	Assault	Assault
	0	0	253	1254	1286	4025	1376	523

### Demographic Trend of Injury Rates by Geographic Characteristics Analysis of Injury Deaths in China, 2004–2010

**Figure 3:**
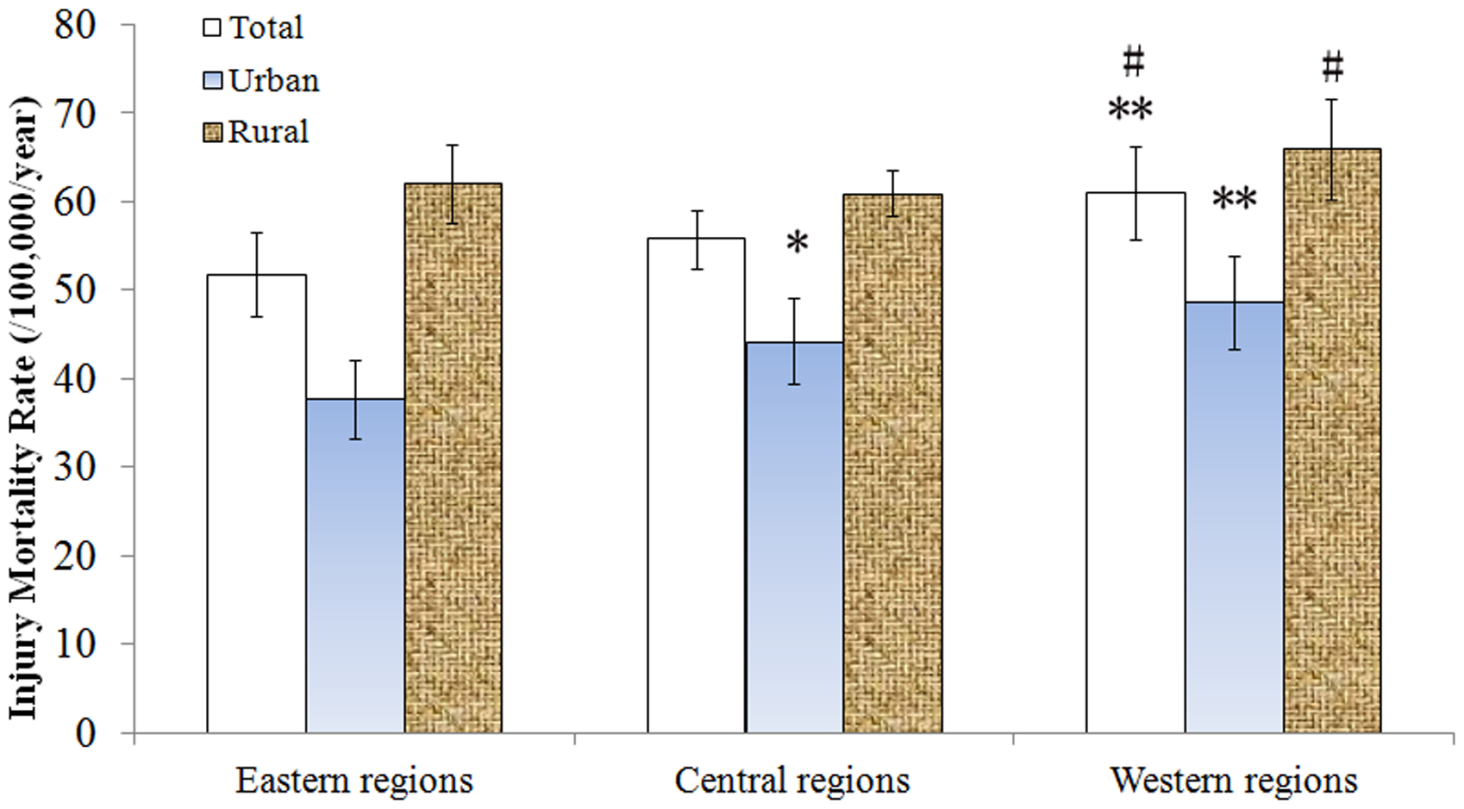
Demographic trend of injury mortality rates (/100,000/year) by geographical divisions in China, 2004–2010. Mortality rates in eastern, central and western regions are compared, respectively. Compared to eastern regions, *p<0.05, **p<0.01; Compared to central regions, # *p*<0.05.

In general, injury mortality rates increased gradually from east to west (Figure 3). The injury mortality rate in eastern region was 51.76±4.74 deaths/100,000/year, which was lower than that of western region (61.04±5.28 deaths/100,000/year) (*p*<0.01). Similarly, the injury mortality rate in central region was 55.79±3.28 deaths/100,000/year, which was also lower than that in western region (*p*<0.05). The injury mortality rate in western urban region was 48.66±5.28, in central urban region 44.25±4.87 and in eastern urban region 37.74±4.38 deaths/100,000/year. The injury mortality rate in western rural region was 66.05±5.67 deaths/100,000/year, which was the highest among all the subgroups differed by geographical divisions.

### Life Lost Caused by Injuries

In this survey, 218,763 persons died of injuries in China throughout the period 2004–2010, but males suffered almost triple the PYLL as females did. Injuries caused 5,036,969 PYLL in males and 1,949,464 PYLL in females. For each fatal injury, AYLL was 31.14 years in males and was 34.20 years in females, respectively (Table 7).

**Table 7:** Person Years of Life Lost and Average Years of Life Lost of injury by gender in China, 2004–2010.

Year	PYLL (person years)			AYLL (years)		
	Male	Female	All	Male	Female	All
2004	798,621	333,444	1,132,065	31.95	34.96	33.46
2005	780,859	318,894	1,099,753	31.77	34.92	33.34
2006	619,903	238,028	857,931	31.48	34.09	32.79
2007	710,180	264,718	974,898	31.34	34.21	33.78
2008	715,094	275,448	990,542	30.73	33.97	32.35
2009	714,550	263,665	978,215	30.59	34.11	32.35
2010	697,762	254,266	952,028	30.12	33.15	31.64
Total	5,036,969	1,949,464	6,985,432	31.14	34.20	32.67

## Discussion

According to World Health Organization (WHO) prediction, by the year 2020, injuries will be responsible for even more morbidity, mortality and disability, with significant socioeconomic impact on the developing countries [14]. As a developing county, it is essential for China to measure injury losses for the productive working population [13]. This study investigated the injury deaths in China from 2004 to 2010 and found important prototypes, disparities and trends in injury mortality rates and patterns by the criteria of urban/rural residency, gender, age and geographic location. Furthermore, this report also provided scientific evidences for the strategic policies and blueprints designed to restrain injury deaths in future.

Our results showed that about 10% of all deaths had resulted from injury-related causes, and 7.5% occurred in rural China, which was lower than the rate 8.9% in rural South Africa 2000–2007 [15]. The major finding of our research underscored the facts that males in the rural areas had experienced highest injury mortality rates, and that there was unquestionable urban-rural disparity in overall injury mortality, with urban residents bearing less risk of injury death than rural residents did. The rural rate was 32.83% higher than the urban rate, which was consistent with the results reported by Jiang et al, (2011), who found a high injury mortality rate in rural males of juvenile and young adult age groups (15–34) in Tianjin, China 1999–2006 [16]. Other studies have also identified higher injury mortality rates in rural areas compared to urban locations in most developing and developed countries [17], [18]. During the study period of this report, however, injury mortality rates in males and in rural areas remained high, a phenomenon might relate to high-risk occupations engaged by males, poor working condition and undeveloped medical aid system in rural areas. Most of the high risk occupations are mainly undertaken by males in China, such as drivers, coal miners, loggers, fishermen, construction workers, skyscraper windows cleaners and so on. In addition, rural residents endure more law violations and high-risk behaviors than their fellow urban residents do, such as driving after alcohol consuming, driving without a license, and violating pedestrian and bicycle traffic rules [19], [20]. Another important underline factor is that the education level of rural population is generally lower than that of urban population. A recent study has suggested that fall-related injuries were inversely related to victims' educational level [21]. Interestingly, similar urban-rural disparity in injury mortality has also been reported in the United States [22], Australia [23] and Norway [24]. Explanations for higher rural rate of injury death in the United State include case fatality rate in motor vehicle crashes, traumatic occupational injuries, drowning, residential fires, and suicide [22]. In Australia, machinery, firearms, being struck by/struck against, fire and burns, natural and environmental factors, motor vehicle crashes and interpersonal violence have been cited as contributing factors [23]. As in Norway, rural victims seem to be younger, die mainly at the site of injury, and from road traffic crashes [24]. By contrast, in Ghana the injury-related mortality was slightly higher in the urban (83 per 100,000) than that in the rural area (53 per 100,000) [25]. Difference in urbanization, socioeconomic development, and motorization may partly account for variations in nationwide urban-rural disparities in different reports on injury mortality [20]. It was gratified that during the period 2004–2010 in China, a welcome tendency of a decline of injury mortality rates was observed over the years in all males, females and urban resident groups. A slight decline of injury mortality rate is encouraging and hopeful, albeit data in rural China are not statistically significant.

According to the analysis of the 2004–2010 National Injury Mortality Surveillance data, the top six injury death causes involved road traffic crashes, suicide, fall, drowning, accidental poisoning and assault. The most worrying tendency was the high injury mortality rate caused by traffic crashes, being 1.5–15 times higher than other rates resulted from the rest leading causes of fatal injuries. Road traffic crashes were responsible for about one third of all injury deaths, and the traffic injury mortality rate, was 17.39–20.90 deaths/100,000/year during the study period. For rural males, this rate skyrocketed to 34.35±1.86 deaths/100,000/year, higher than any result other subgroups by urban/rural areas, and much higher than that of urban males. In early 2009, China became the world's largest automobile market, surpassing the United States for the first time in total car sales [26]. Corresponding to the rapid growth in road construction and number of vehicles, road traffic injury has become a serious public health concern in China [11]. Although in 2007, the Development and Research Center of Chinese State Council published a report that identified several major shortfalls that were responsible for road traffic injury in China, including lack of safety standards in road construction, and ambiguities in road safety laws and regulations [27]. In particular, the mortality rate caused by road traffic crashes failed to show any tendency of decline. In summary, careless male drivers in rural areas, vehicle overloading, speeding, drink/drunk driving, obsolete safety regulations, limited access to urgent care and/or poor quality of care in the rural areas imply lift up the risks of traffic injury mortality.

Overall, the number one leading cause of injury death was road traffic crashes, followed by suicide and falls. Suicide injuries emerged as the most common injury category for rural areas. During 2004–2010, suicide injury mortality rate for rural males and females was 13.08±2.07 and 11.42±1.93 deaths/100,000/year, respectively. Although the rate in rural females was lower than that of rural males, suicide has become the number one causes of injury mortality for rural females. In this report, the suicide rate of rural females was 1.76 times higher that of urban females, distinctively different from the panoramas in high-income countries, where differences in urban and rural suicide rates are marginal and vary only by locations [28]. China and India are the two biggest political entities where absolutely top numbers of suicides exist [29]. Young women in rural areas in both countries are at especially high risk of dying by committing suicide, accounting for about a third of all mortality cases of the age group; and the common methods of suicide is self poisoning with pesticides [30]. In China, the demographic, social, and psychological issues have been assumed as universal risk factors for suicide, much different from the findings in the high-income countries where suicidal behavior is almost always associated with a mental illness, such as depression [31]. Furthermore, there are no strong religious or legal prohibitions against suicide, so people with serious mental disorders or chronic life stressors might commit suicide to relieve themselves from misery and emotional burden [32]. Shan et al have indicated that reasons for attempted suicide in Shanghai Municipality often appear to be related to family conflicts and unemployment [33]. In this issue, residents in urban and rural areas differ in the time needed to reach emergency departments, the availability of health care organizations, and the capacity of health care practitioners [20].

This report also portrays a detailed breakdown of causes of fatal injuries by age, which offers important information to set prevention measures across all age groups. For instance, preventing infants in the zero-year-old age group from drowning is very important, but it is even more imperative for children in the 5–14 year-old age group where deaths resulted from drowning actually exceeds the number from road traffic crashes.

Whilst traffic crashes remain the primary prevention target for people across the 1–4, 15–44 and 45–64 year-old age groups, Prevention of falls demands special attention for seniors in the group of 65 year-old and beyond. Without pre-symptoms, senior citizens may fall over road curbs, loopholes, staircases, etc, resulting in injuries or deaths.

Sadly, suicide becomes the second leading cause of fatal injury for persons in the groups of 15 year-olds and beyond. According to China CDC, the suicide rate in this country is 22.23 out of every 100,000 people. More than 30% of suicides worldwide occur in China, illustrating the largest suicide problem in the world [34]. In China, about 40% of suicides directly result from anxiety, alcohol abuse, intolerable working conditions and highly toxic pesticides abuse. Table 6 is, therefore, a constructive tool for designing injury prevention programs in different age groups.

Economic status has been documented to be an important determinant of injury. In this study, those in western rural regions with the lowest income went through a higher risk of injury incidence. However, Ma et al demonstrated that those in both the lowest- and highest-income brackets were at a higher risk of injury incidence [35]. Another study in America showed that, in the multivariate models, income and education were largely unrelated to overall injury morbidity [36]. Death and potential years of life lost lead to huge economic losses to individuals, families and the society. In this report, the PYLL associated with injury was significantly higher in males than that in females, China 2004–2010. However, the AYLL was significantly lower in males than that in females, 31.14 versus 34.20 years. This phenomenon may again be due to the facts that males more often participate in high-risk professions and engage in more emergent and dangerous activities than females do, and that the life expectancy for females is generally longer than males.

Our results show that lack of awareness of traffic regulations, poor road conditions, suicide and drowning are leading risk factors of fatal injuries in China. Males, rural area, and western region should be the key targets for injury prevention policies. More psychological counseling and health education should be performed for rural females. Also, there is an earnest need for injury prevention policies for elderly residents, regarding falls and suicide. Moreover, it is critically important to develop policies and programs that can deliver effective measures in the high risk populations and areas.

## Data and Methods

### Data Source

Data of this study obtained from Death Surveillance Data Sets, which were summarized in the National Disease Surveillance Point (DSPs) system and compiled by Chinese Center for Disease Control and Prevention (CDC). The DSPs system covers 1% representative sample of China's population in 31 provinces, autonomous regions and municipalities, which is epitomizes the characteristics of Chinese population. Causes of death were classified according to the International Classification of Diseases-10^th^ Revision (ICD-10) (World Health Organization, 1992). We disaggregated the data by age (≤0, 1–4, 5–14, 15–24, 25–34, 35–44, 45–64, ≥65+) [37], gender and major geographic focus (East, Central, West). We also portrayed rate trends for the six leading causes of injury throughout the study period. The age-adjusted mortality rates were cited from the DSPs, whose direct standardization procedure employed China standard population of the year 2000 as the referent.

### Geographical Divisions and Population Geography

**Figure 4:**
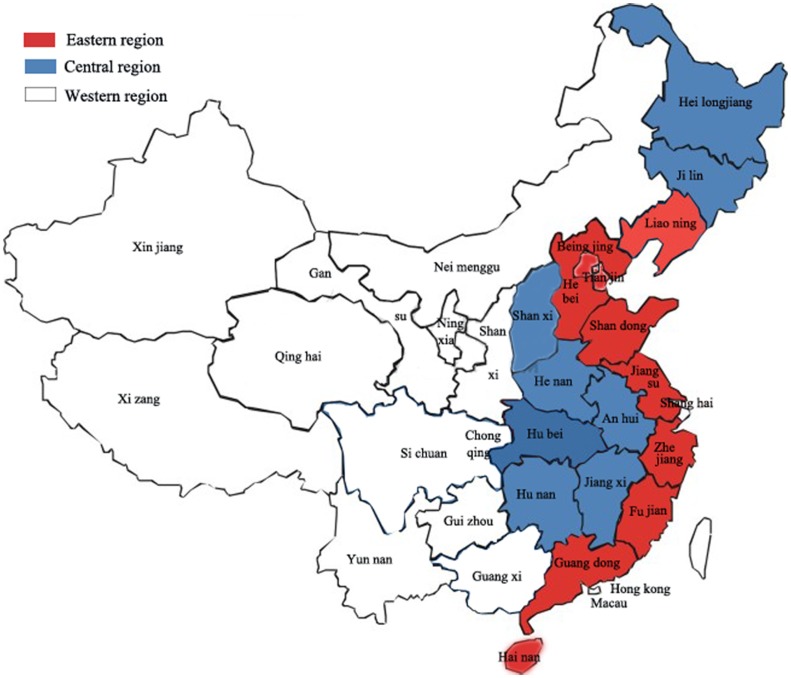
Map of China with geographical divisions. A map shows the locations of eastern, central and western regions divided by geographical position. Red: eastern region; blue: central region; write: western region.

According to the classification of the National Statistics Bureau, China is divided into 22 provinces, 4 municipalities (Beijing, Shanghai, Tianjin and Chongqing), 5 autonomous regions (Guangxi, Ningxia, Xizang, Xinjiang and Neimenggu), and 2 special administration regions (Hong Kong and Macau). Moreover, according to the first national economic census, China is divided into three regions: eastern, central and western regions (Figure 4). More than half of the secondary and tertiary industry units are concentrated in the eastern regions, and the number of units displays a decreasing trend from east to west. There are economic gaps between the eastern and central and western regions in China. The economy in the eastern region of China is considerably stronger than those in other regions [38], [39].

### Data Analysis

Epidata 3.1 was used for data input, as statistical software SPSS 20.0 (SPSS, Inc., Chicago, IL, USA) was used for data analysis. Rates and proportions were the main indexes of the study. Age- and gender-specific mortality rates per 100,000 person-years were calculated using the methods suggested by Rothman and Greenland [40]. For comparison purposes, the age- and gender-specific mortality rates by category of injury were also calculated as well. A chi-square test was used for examining the eastern, central and western regions differences in injury mortality. When the assumptions for the chi-square test were violated, it was replaced by Fisher's exact test. Time trends were calculated using linear regression analysis. *p*<0.05 was selected as the statistically significant level.

Formula and calculation [35]: Person Years of Life Lost (PYLL) = 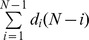, Average Years of Life Lost (AYLL) = 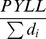. I = age at death; d_i_ = number of deaths at age i; N = upper cut-off age, 71 for males and 75 for females were adopted, respectively.
